# ABO blood type and the risk of cancer – Findings from the Shanghai Cohort Study

**DOI:** 10.1371/journal.pone.0184295

**Published:** 2017-09-07

**Authors:** Joyce Yongxu Huang, Renwei Wang, Yu-Tang Gao, Jian-Min Yuan

**Affiliations:** 1 Division of Cancer Control and Population Science, University of Pittsburgh Cancer Institute, Pittsburgh, Pennsylvania, United States of America; 2 Department of Epidemiology, University of Pittsburgh, Pittsburgh, Pennsylvania, United States of America; 3 Department of Epidemiology, Shanghai Cancer Institute, Shanghai Jiaotong University, Shanghai, China; Centro Nacional de Investigaciones Oncologicas, SPAIN

## Abstract

ABO blood type is an inherited characteristic. The associations between ABO blood type and risk of all cancer and specific cancers were examined in a prospective cohort study of 18,244 Chinese men enrolled in 1986. During the 25 years of follow-up, 3,973 men developed cancer including 964 lung cancers, 624 colorectal cancers, 560 gastric cancers, 353 liver cancers, and 172 urinary bladder cancers. Hazard ratios (HR) for all cancer and specific cancers by ABO blood type were calculated using Cox proportional hazards models. Compared with blood type A, blood type B was associated with statistically significant reduced risk of all cancers (HR, 0.91, 95% CI:0.84, 0.99). Both blood types B and AB were associated with significantly lower risk of gastrointestinal cancer and colorectal cancer, respectively. Blood type B was also associated with significantly lower risk of stomach cancer and bladder cancer, while blood type AB was associated with significantly increased risk of liver cancer. By histological type, blood types B and AB were associated with lower risk of epidermoid carcinoma and adenocarcinoma, but were not associated with risk of sarcoma, lymphoma, leukemia or other cell types of cancer. The findings of this study support a role of genetic traits related to ABO blood type in the development of cancers in the gastrointestinal and urinary tracts.

## Introduction

The ABO blood types, discovered by Karl Landsteiner in 1901, are medically the most important blood types. The ABO blood types are defined by carbohydrate moieties displayed on the surface of red blood cells and attached to a protein backbone, known as the H antigen [1]. A simple test for the presence or absence of antigen A or antigen B in the blood can determine an individual’s ABO blood type [2]. Individuals with blood type A have antigen A present but absence of antigen B in their red blood cells whereas those with blood type B have antigen B but no antigen A. People with blood type AB have both antigens A and B present in the red blood cells. In contrast, individuals with blood type O have neither antigen A nor antigen B expressed in their red blood cells [3]. In addition to their expression on the surface of red blood cells, the ABO antigens are highly expressed on the surface of epithelial cells of the gastrointestinal, bronchopulmonary, and urogenital tracts [1]. Alterations in surface glycoconjugates may lead to modifications in intercellular adhesion, membrane signaling, and immunosurveillance, which could have important implications for the development of cancer [1, 4].

Aird et al in 1953 discovered a statistically significant association between blood type A and risk of gastric cancer [5]. This finding has stimulated an immense amount of research that examined the relationship between ABO blood type and risk of cancer, other chronic diseases, and infectious diseases. The results of those early studies drew severe criticisms. The critiques included (1) the use of hospital-based case series that were not representative for the patient population under study, (2) the inappropriate use of a comparison group that comprised blood donors (blood type O could be overrepresented due to the universal donor status), relatives of patients (the similar genetic traits) or other patients who were admitted to the same hospitals for other diseases, and (3) uncontrolled for potential confounding effects on the association between ABO blood type and disease risk. Results from well designed and well executed prospective cohort studies would be required to confirm those early observations [6]. A recent epidemiological study in two large cohorts in the United States found that self-reported ABO blood types were statistically significantly associated with risk of pancreatic cancer [7]. Given that ABO blood types are inherited through genes on chromosome 9q34 [8], a genome-wide association study also noted an association between DNA sequence variants in the *ABO* locus and susceptibility to pancreatic cancer. These findings further support a role of ABO blood type as a risk marker for pancreatic cancer [9].

Epidemiologic data in Asian populations, in particular from population-based prospective cohort studies, are sparse. Most of previous prospective studies only examined the ABO blood type with risk of single cancer site. In the present study, we utilized the data set of a prospective cohort of more than 18,000 men with up to 25 years of follow-up to examine the association between ABO blood type and the risk of all cancer and specific cancers by site or histology. The determination of ABO blood type for each study participant at baseline provided an unique opportunity to test the hypothesis that ABO blood type contributes to subsequent risk of cancer development.

## Materials and methods

### Study population

The Shanghai Cohort Study began in 1986. The study design and subject recruitment has been previously described in details [10, 11]. Briefly, the cohort consisted of 18,244 men (constituting 80% of eligible subjects) enrolled from January 1, 1986 through September 30, 1989, who were between 45 and 64 years of age and resided in one of four small geographically defined communities in Shanghai, China. At enrollment, each participant was interviewed in person by a trained research nurse using a structured questionnaire asking for information on demographic characteristics, history of tobacco and alcohol use, adult dietary habits, and medical history. Following the completion of the interview, participants were asked to provide a 10-mL blood sample and a single-void urine specimen. The Institutional Review Boards at the Shanghai Cancer Institute and the University of Pittsburgh had approved the Shanghai Cohort Study. All procedures performed were in accordance with the ethical standards of these IRBs and with the 1964 Helsinki declaration and its later amendments.

The ABO blood type was determined at the time of baseline blood draw using a standard test for the presence of ABO antigens in subject’s whole blood at the blood draw station (i.e., ABO forward grouping method) [2]. The test result of the ABO blood type was recorded in the study questionnaire at baseline.

### Identification of outcome events

Follow-up of the cohort for incident cancers and deaths has been ongoing through multiple means that were previously described [10]. In addition to annual in-person follow-up interviews to all surviving cohort members, we performed periodically record linkage analysis of all study participants with the databases of the Shanghai Cancer Registry and Shanghai Municipal Vital Statistics Office, respectively. As of December 31, 2013, the cut-off date for the present analysis, only 670 (3.7%) original cohort members were lost to follow-up and additional 598 (3.3%) refused to our annual follow-up interviews since the beginning of this cohort study. For these subjects, we searched the cancer registry and death index databases to ascertain their cancer diagnosis and vital status, and cause and date of death. Therefore, the follow-up for incident cancer and death for the entire cohort was virtually completed.

When we identified a new cancer patient by self-reports or reports of the-next-of-kin of the study participants who died, or through linkage analysis with the cancer registry database, we already had the permission granted by the study subject to review his medical records and pathology reports. During the review of these records, a research nurse completed a medical record abstract form that recorded information on cancer diagnosis including site, date of initial diagnosis, basis of diagnosis, and histology of cancer, if available. Both topography (site) and morphology (histology) of all cancer cases were coded according to the World Health Organization’s International Classification of Diseases 9^th^ version (ICD-9) and ICD for Oncology (ICD-O), respectively. Besides groups of cancer cases by system (i.e., digestive or respiratory system) or individual site, we further classified cases by histology in a standard way according to the previous description [12]. This system classifies histological diagnoses of cancers into carcinoma (ICD-O: 801–867), lymphoma (ICD-O: 959–971), sarcoma and other soft tissue tumors (ICD-O: 868–871, 880–892, 899, 904, 912–934, 937, 954–958), leukemia (ICD-O: 980–994), and other specified or unspecified types of cancer. For carcinoma, this system further classified cancer cases into epidermoid carcinomas (ICD-O: 805–813), adenocarcinoma (ICD-O: 814, 816, 818–822, 825–850, 952–855, 857), other specified (ICD-O: 803–804, 815, 817, 823, 824, 851, 856, 867) and unspecified carcinomas (ICD-O: 801–802) [12].

### Statistical analysis

The present analysis included 18,069 individuals after we excluded 146 subjects with a history of cancer at baseline, 5 cancer cases with missing information on diagnosis date (2 lung cancer, 1 esophagus cancer, 1 colon cancer, and 1 gallbladder cancer), and 24 with unknown ABO blood type. The χ^2^ test and the analysis of variance (ANOVA) methods were used to compare the distributions of selected demographic characteristics and lifestyle factors at baseline by ABO blood type.

For each individual, person-years of follow-up were calculated from the date of recruitment to the date of cancer diagnosis or death, the date of last known vital status for those lost to follow-up, or the date of the last annual follow-up interview, whichever occurred first. The Cox proportional hazards regression models were used to examine the associations between ABO blood type and risk of all cancer combined or groups of common cancers by site or histology. The magnitude of the association was assessed by the hazard ratio (HR) and its 95% confidence interval (CI) and *P* value. All Cox regression models were adjusted for age at baseline interview, year of baseline interview, level of education (no formal school or primary school, junior middle school, senior middle school, or college or above), body mass index (kg/m^2^), cigarette smoking status (never, former, or current smokers), and alcohol intake (nondrinkers, <3 drinks, or 3 or more drinks per day).

Statistical analyses were carried out using SAS software version 9.3 (SAS Institute, Cary, NC). All *P*-values reported are two-sided. *P’s* less than 0.05 were considered to be statistically significant.

## Results

Among 18,069 original cohort participants with available ABO blood types, 32% were blood type O, 31% type A, 27% type B, and 10% type AB. The distributions of age, body mass index, level of education, smoking status and the number of cigarettes consumed per day or over lifetime, and alcohol intake were comparable across different ABO blood types (**Table 1**).

**Table 1:** Baseline demographic and lifestyle characteristics by ABO blood type, Shanghai Cohort Study 1986–2013.

	ABO blood type				*P*^*a*^
	A	B	AB	O	
No. of persons	5,586	4,891	1,890	5,702	
Age at interview (year), mean (SD)	55.7(5.7)	55.7 (5.8)	55.9 (5.7)	55.8 (5.8)	0.329
Body mass index (kg/m^2^), mean (SD)	22.2 (3.0)	22.2 (3.0)	22.2 (3.0)	22.1 (2.9)	0.496
Level of education, n (%)					
Primary school or below	1596 (28.6)	1390 (28.4)	557 (29.5)	1599 (28.0)	
Secondary school	2622 (46.9)	2266 (46.3)	898 (47.5)	2673 (46.9)	0.581
College or above	1368 (24.5)	1235 (25.3)	435 (23.0)	1430 (25.1)	
Smoking status, n (%)					
Never smokers	2369 (42.4)	2078 (42.5)	794 (42.0)	2497 (43.5)	
Former smokers	372 (6.7)	323 (6.6)	151 (8.0)	368 (6.5)	0.312
Current smokers	2845 (50.9)	2490 (50.9)	945 (50.0)	2855 (50.0)	
Among ever smokers					
No. of cigarettes/day, mean (SD)	16.1 (8.2)	16.3 (8.3)	16.1 (8.2)	16.3 (8.3)	0.764
No. of years of smoking, mean (SD)	29.7 (10.8)	29.3 (10.6)	29.7 (10.7)	29.7 (10.6)	0.543
No. of pack-years of smoking, mean (SD)	25.0 (16.7)	25.0 (16.6)	25.0 (16.6)	25.5 (16.9)	0.827
Alcohol drinking status (%)					
Non-drinkers	3178 (56.9)	2783 (56.9)	1070 (56.6)	3338 (58.5)	0.204
Drinkers	2408 (43.1)	2108 (43.1)	820 (43.4)	2364 (41.5)	
Among drinkers					
No. of drinks/day, mean (SD)	2.4 (2.4)	2.4 (2.4)	2.4 (2.4)	2.3 (2.6)	0.579
No. of years of drinking, mean (SD)	26.3 (13.0)	25.6 (13.2)	26.2 (13.0)	25.3 (13.3)	0.115
Ethanol intake over lifetime (kg), mean (SD)	329 (423)	323 (420)	326 (411)	306 (483)	0.597
*H*. *pylori* antibody serologic status, n (%)^b^					
Negative	45 (14.1)	55 (19.2)	17 (18.3)	53 (16.9)	0.390
Positive	274 (85.9)	231 (80.8)	76 (81.7)	261 (83.1)	
HBsAg serologic status, n (%)^c^					
Negative	338 (89.9)	295 (91.1)	110 (90.2)	398 (90.3)	0.964
Positive	38 (10.1)	29 (9.0)	12 (9.8)	43 (9.8)	

Abbreviations: n, number; No., number; SD, standard deviation

^a^ Two-sided *P*’s were derived from analysis of variance (ANOVA) for continuous values or chi-square test for categorical or nominal values.

^b^ Based on 1,012 cohort members with available measurement of *Helicobactor pylori* (*H*. *pylori*) who remained free of cancer by the latest follow-up.

^c^ Based on 1,263 cohort members with available measurement of hepatitis B surface antigen (HBsAg) who remained free of cancer by the latest follow-up.

After 25 years of follow-up, the cohort accumulated a total of 355,797 person-years and 3,973 incident cancer cases by the end of 2013. The cancer of digestive system was the most frequent diagnosis that accounted for 51% of total cancer cases, followed by respiratory system (26%), and genitourinary system (13%). Lymphoma, leukemia and sarcoma combined together accounted for 4% of total malignancies. Cancer of the skin, bone and connective tissue were relatively rare (accounting for only 2%). The remaining 4% malignancies belonged to other or unspecified sites. By individual sites, cancer of the lung was the most common cancer diagnosis (accounting for 24% of total cancers), followed by colorectum (16%), stomach (14%) and liver (9%). The esophageal, pancreas, prostate and urinary bladder cancer each accounted for approximately 6% of total cancer cases (**Table 2**). By histology, carcinoma accounted for 60% of total histologically confirmed cancer cases while sarcomas, lymphoma and leukemia altogether accounted for 4%. The remaining 36% of cancer cases consisted of cases with other histology types or no histological confirmation. Among carcinomas, 66% were adenocarcinomas, 25% were epidermoid carcinomas, and the remaining 9% were other or unspecified carcinomas (**Table 2**).

**Table 2:** Number of cancer cases by ABO blood type, Shanghai Cohort Study 1986–2013.

	ABO blood type				Total
	A	B	AB	O	
No. of person-years	109,201	96,975	36,925	112,696	355,797
No. of all cancer cases	1,284	1,036	406	1,247	3,973
By cancer site (ICD-9 codes)					
Digestive system (140–159)	668	519	204	625	2,016
Gastrointestinal tract (140–154)	486	351	126	442	1,405
Esophagus (150)	44	43	10	40	137
Stomach (151)	199	131	58	172	560
Colorectum (153–154)	221	155	51	197	624
Liver (155)	101	87	50	115	353
Pancreas (157)	53	47	15	47	162
Respiratory system (160–165)	320	278	112	323	1,033
Lung and trachea (162)	302	256	104	302	964
Bone/connective tissue/skin (170–173)	24	24	3	18	69
Genitourinary organs (185–189)	172	125	51	160	508
Prostate (185)	86	69	25	72	252
Urinary bladder (188)	59	34	19	60	172
Lymphoma/multiple myeloma/leukemia (200–208)	54	45	15	57	171
All other or unspecified sites	46	45	21	64	176
By cancer histology ^a^					
Carcinoma	802	595	227	767	2,391
Epidermoid carcinoma	201	160	52	195	608
Adenocarcinoma	537	397	155	494	1,583
Sarcoma/lymphoma/leukemia	58	51	14	46	169
All other/unspecified neoplasm	424	390	165	434	1,413

Abbreviations: ICD, International Classification of Diseases; No., number

^a^ See the ICD-O codes in the Materials and Methods section.

Compared with blood type A, blood type B was associated with a statistically significant lower risk of all cancers (**Table 3**). The incidence of cancers of the gastrointestinal tract was statistically significantly different among different ABO blood types. Compared with blood type A, individuals with non-A blood types were at an approximately 20% lower risk of cancer of the gastrointestinal tract (HR = 0.83, 95% CI = 0.75–0.93, *P* = 0.001), stomach cancer (HR = 0.80, 95% CI = 0.67–0.95, *P* = 0.013), and colorectum cancer (HR = 0.80, 95% CI = 0.68–0.94, *P* = 0.008). For blood type B, the hazard ratio of developing stomach and colorectal cancer was 0.75 (95% CI = 0.76–0.94, *P* = 0.009) and 0.78 (95% CI = 0.64–0.96, *P* = 0.020), respectively. Blood type AB was also associated with significantly reduced risk of cancers in the gastrointestinal tract (HR = 0.76, 95%CI = 0.63–0.93, *P* = 0.007) or the large intestine (HR = 0.68, 95%CI = 0.50–0.92, *P* = 0.013). Conversely, when compared with blood type O, individuals with blood type A had a statistically borderline significant higher risk of stomach cancer (HR = 1.21, 95% CI = 0.98–1.48, *P* = 0.070) and colorectal cancer (HR = 1.18, 95% CI = 0.97–1.43, *P* = 0.097) (**Table 3**).

**Table 3:** Hazard ratios (95% confidence intervals) for cancer associated with ABO blood type Shanghai Cohort Study 1986–2013.^a^

	ABO blood type			
	A	B	AB	O
All cancer	1.00 (referent)	**0.91 (0.84–0.99)** ^**b**^	0.93 (0.83–1.04)	0.93 (0.86–1.01)
By cancer site				
Digestive system	1.00 (referent)	**0.88 (0.78–0.98)** ^**b**^	0.90 (0.77–1.05)	0.90 (0.81–1.00)
Gastrointestinal tract	1.00 (referent)	**0.81 (0.71–0.93)** ^**b**^	**0.76 (0.63–0.93)** ^**b**^	0.88 (0.77–1.00)
Esophagus	1.00 (referent)	1.13 (0.74–1.72)	0.67 (0.34–1.33)	0.90 (0.58–1.37)
Stomach	1.00 (referent)	**0.75 (0.60–0.93)** ^**b**^	0.86 (0.64–1.15)	0.83 (0.68–1.02)
Colorectum	1.00 (referent)	**0.78 (0.64–0.96)** ^**b**^	**0.68 (0.50–0.92)** ^**b**^	0.85 (0.70–1.03)
Liver	1.00 (referent)	0.98 (0.73–1.30)	**1.45 (1.04–2.04)** ^**b**^	1.10 (0.84–1.43)
Pancreas	1.00 (referent)	1.00 (0.67–1.47)	0.83 (0.47–1.47)	0.85 (0.57–1.26)
Respiratory system	1.00 (referent)	1.00 (0.85–1.17)	1.03 (0.83–1.27)	0.97 (0.83–1.14)
Lung and trachea	1.00 (referent)	0.97 (0.82–1.15)	1.01 (0.81–1.26)	0.96 (0.82–1.13)
Bone/connective tissue/skin	1.00 (referent)	1.12 (0.63–1.97)	0.37 (0.11–1.24)	0.72 (0.39–1.33)
Genitourinary organs	1.00 (referent)	0.80 (0.64–1.01)	0.88 (0.71–1.09)	0.88 (0.64–1.20)
Prostate	1.00 (referent)	0.87 (0.64–1.20)	0.86 (0.55–1.34)	0.79 (0.58–1.08)
Urinary bladder	1.00 (referent)	**0.64 (0.42–0.98)** ^**b**^	0.95 (0.57–1.59)	0.96 (0.67–1.38)
Lymphoma/multiple myeloma/leukemia	1.00 (referent)	0.93 (0.63–1.38)	0.82 (0.46–1.46)	1.01 (0.69–1.46)
All other or unspecified sites	1.00 (referent)	1.10 (0.73–1.66)	1.36 (0.81–2.27)	1.35 (0.92–1.97)
By cancer histology				
Carcinoma	1.00 (referent)	**0.84 (0.75–0.93)** ^**b**^	**0.83 (0.72–0.97)** ^**b**^	0.92 (0.83–1.02)
Epidermoid carcinoma	1.00 (referent)	0.91 (0.74–1.12)	0.76 (0.56–1.03)	0.94 (0.77–1.14)
Adenocarcinoma	1.00 (referent)	**0.83 (0.73–0.94)** ^**b**^	0.85 (0.71–1.02)	0.88 (0.78–1.00)
Sarcoma/lymphoma/leukemia	1.00 (referent)	0.98 (0.67–1.43)	0.72 (0.40–1.28)	0.76 (0.51–1.11)
All other/unspecified neoplasm	1.00 (referent)	1.04 (0.91–1.20)	0.98 (0.86–1.12)	1.14 (0.95–1.36)

^a^ Adjusted for age at baseline, body mass index, year of interview, level of education, smoking status (never, former, current), and alcohol intake (nondrinker, <3 drinks/day, 3+ drinks/day).

^b^ Two-sided P < 0.05.

Subjects with blood type AB were at increased risk of liver cancer with a statistically significant 42–45% higher risk of liver cancer **(Table 3)**. HR for liver cancer for individuals with blood type AB was 1.45 (95% CI = 1.04–2.04, *P* = 0.028) when comparing with blood type A or 1.42, (95% CI = 1.05–1.91, *P* = 0.023) when comparing with non-AB blood type (i.e., blood types A, B, and O combined).

In the present study, blood type AB and O were associated with reduced risk of developing pancreatic cancer compared with blood type A. Compared with blood type A, individuals with blood types AB and blood type O experienced a 17% and 15% lower risk of pancreatic cancer, respectively, but none of them reached statistical significance (**Table 3**).

Blood type B was associated with reduced risk of urinary bladder cancer. Compared with blood type A, subjects with blood type B had a statistically significant 36% lower risk of urinary bladder cancer (HR = 0.64, 95% CI = 0.42–0.98, *P* = 0.040). Individuals with blood type AB or O had bladder cancer risk comparable with blood type A (**Table 3**). ABO blood type was not associated with risk of cancer of the lung, prostate, skin, bone, and connective tissue (**Table 3**).

When cancer cases were classified by histological type, blood type B and AB were associated with a statistically significant 15~17% lower risk of carcinoma or adenocarcinoma compared with blood type A (**Table 3**). Similarly, compared with blood type A, hazard ratio for carcinoma were 0.84 (95% CI = 0.75–0.93, *P* <0.001) for blood type B. When compared with blood type A and O combined (i.e., absence of antigen B), individuals with blood type B and AB (i.e., presence of blood antigen B) experienced a statistically significant 13% lower risk of carcinoma (HR = 0.87, 95% CI = 0.80–0.95, *P* = 0.001) and a 11% lower risk of adenocarcinoma (HR = 0.89, 95% CI = 0.80–0.98, *P* = 0.025). Blood types AB and O were associated with statistically non-significant 24~28% lower risk of sarcoma, lymphoma, or leukemia combined compared with blood type A.

Analyses were repeated after excluding individuals who developed cancer within the first two years of blood draw and the corresponding person-years of follow-up. In general, the results remained unchanged except for that the inverse association between blood type AB and risk of adenocarcinoma became stronger and statistically significant (HR = 0.81, 95% CI = 0.67–0.98, P = 0.028) (**S1 Table**). Further adjustment for history of diabetes and high blood pressure did not materially change the association between ABO blood type and cancer risk (data not shown).

## Discussion

This prospective study of a cohort of middle-aged or older Chinese men demonstrates a lower risk of all cancer for blood type B, as well as lower risk for gastrointestinal cancers including stomach and colorectal cancer for blood types B and AB than blood type A. In contrast, the present study showed a higher risk of liver cancer for blood type AB and lower risk of urinary bladder cancer for blood type B. These findings are consistent with the notion that besides red blood cells, the ABO blood type antigens are expressed in epithelial cells of gastrointestinal and urinary tract, and suggesting a role of ABO blood type in the development of epithelial cancers in the gastrointestinal and urinary tracts.

For several decades, a role of ABO blood type antigens in the development of cancer has been suspected. Since the initial reports of Aird et al of the association of peptic ulcer and gastric carcinoma with ABO blood types [5, 13], a voluminous literature has accumulated on these topics, and it has been summarized previously [6, 14]. Those previous studies, mostly conducted in Western populations, consistently showed an approximately 20% excess risk of gastric cancer in individuals with blood type A. Data from Chinese population are sparse. A cross-sectional study in northern China found that blood type A was associated with statistically significant 30–40% higher risk of intestinal metaplasia or gastric dysplasia, the most advanced pre-malignant lesions in the stomach, in people with blood type A than in those with other blood types [15]. A recent prospective cohort study in a Taiwanese population showed that blood type A was associated with a 38% increased risk of stomach cancer [16]. The present study found a 26% increased risk of gastric cancer for blood type A as compared with blood types B and O (HR = 1.26, 95% CI = 1.06–1.51, *P* = 0.011), consistent with findings of earlier studies in Chinese as well as in Western populations.

The mechanism for the association between ABO blood type and gastric cancer is not completely understood. Experimental studies have found that the specific blood type antigen (e.g., Lewis^b^), the product of blood type genes at 19q13, mediates the attachment to human gastric mucosa of *H*. *pylori*, a causative agent in chronic active gastritis, gastric and duodenal ulcers, and gastric adenocarcinoma [17]. Human epithelial cells of type O individuals bound significantly more *H*. *pylori* and had greater inflammatory responses to *H*. *pylori* than did cells of persons with other blood types [18, 19]. *H*. *pylori* bacteria could not bind to gastric tissue that lacks the Lewis^b^ antigen [17, 20]. These data suggest that the availability of *H*. *pylori* receptors might be reduced in people with blood type B and AB phenotype as compared with blood type O. These experimental studies have provided strong biological explanations for the higher prevalence of peptic ulcer among individuals with blood type O than type A [6]. In contrast, an excessive risk of gastric cancer associated with blood type A in the present study as well as many previous studies strongly implicates a different mechanism. The controversy of increased risk of gastric cancer among individuals with blood type A while a high peptic ulcer risk among those with blood type O was also observed in another cohort study among Scandinavian populations [21]. Further studies are warranted to delineate the mechanism between ABO blood type and risk of gastric cancer.

In addition to the stomach, we extended our analysis to the entire gastrointestinal tract. Similar to gastric cancer, blood type A individuals experienced statistically significantly increased risk of colorectal cancer compared to those with non-A blood type. These findings are consistent with previous summary report in Western populations [6] and a prospective study in Taiwan [22]. Experimental studies have demonstrated that the expression of ABO blood type antigens on colon tumor cells, and the cell proliferation and motility are highly associated with the expression of blood type antigen A [23, 24]. These data suggest a direct involvement of ABO blood type antigens in the development and metastasis of colorectal cancer.

The present study also showed an increased incidence of pancreatic cancer among subjects with blood type antigen A or B compared with those who were lack of these antigens (i.e., blood type O). Although none of these associations reached statistical significance due to relatively small number of pancreatic cancer cases, these findings were consistent with findings of the Nurses’ Health Study and Health Professionals Follow-up Study [7].

The present study noted several novel associations between ABO blood type and risk of cancer of the liver and urinary bladder. Subjects with blood type AB had a statistically significant 45% increased risk of liver cancer. In laboratory investigations, patient-derived hepatocellular tumor cells express blood type antigens on their cell surface and have different patterns of expression than cells in adjacent nontumorous liver tissues [25, 26], indicating a potential role of the ABO blood type antigens in the malignant transformation of hepatocytes. A strong association between plasma alkaline phosphatase, a liver enzyme, and genetic polymorphisms within the *ABO* locus on chromosome 9q34[8], further supports the biologic link for ABO blood type to the liver function [27]. Alternations in the host inflammatory state due to ABO blood type antigens may provide a further mechanism to explain an association between blood type and liver cancer risk. Chronic infection with hepatitis B is a primary causal factor for liver cancer in this population [28]. A genome-wide association study has noted a direct link of single nucleotide polymorphisms at the *ABO* locus to serum levels of tumor necrosis factor-alpha [29], an inflammatory cytokine known to modulate risk of liver cancer [30]. Although biologic evidence is strong, this novel statistical association between blood type AB and liver cancer risk needs to be replicated and confirmed in other populations.

Another novel observation of the present study is the reduced risk of urinary bladder cancer for men with blood type B relative to type A. ABO blood type antigens are constitutively expressed on urothelial cells. Studies have found that large proportion of transitional epithelia of bladder carcinoma had either reduced expression of blood type antigen A or loss of *A* allele [31, 32]. A reduction in the expression of antigen A was correlated with the invasiveness of the malignancies [33]. These pathological findings suggest that the determinants of ABO blood type antigens may play a critical role in the development of urinary bladder cancer. Future studies are warranted to examine the role of the determinants of ABO type antigens or closely linked genetic variations in the development of bladder cancer.

In the present study population of men aged 45–64 years at blood draw, 32% were blood type O, 31% blood type A, 27% blood type B, and 10% blood type AB. The corresponding frequencies in southern Chinese (n = 76,039) were 26%, 34%, 30%, and 10% [34]. In American Chinese (n = 37,822), most immigrants originated from southern China, the prevalence of blood types O, A, B, and AB were 42%, 27%, 25%, and 6%, respectively [35]. Both sets of ABO phenotype frequencies were derived from blood donors, which may explain the difference in the blood type prevalence between the populations of our study and of earlier studies.

The present study has several strengths. The blood type was determined using a standard agglutinin assay for all study participants at recruitment, which avoided inaccurate ABO blood type by self-reports. The prospective design of the study minimized potential selection bias on the inclusion of study subjects due to disease status. A comprehensive questionnaire for collection of potential confounders and demographic information at baseline allowed for controlling for potential confounding effect on the association between the ABO blood type and cancer risk. The follow-up of the cohort participants for incidence of cancer and death was virtually complete, minimizing the potential selection bias due to the loss of follow-up. Relatively large number of incident cancer cases provided sufficient statistical power to examine the association between the ABO blood type and risk of cancer by sites and histology. Our post hoc analysis revealed that the present study had an 80% statistical power to detect a minimal hazard ratios of 1.13 for total cancer, 1.16 for total carcinoma, 1.22 for gastrointestinal cancer, 1.37 for stomach cancer, and 1.34 for colorectal cancer associated with blood type B comparing blood type A. The present study shows higher HRs that these values, suggesting a sufficient statistical power.

The main limitation of the present study is the relatively small sample size of certain specific cancers including pancreatic cancer, which otherwise would provide more definitive results. Another drawback of the present study was multiple comparisons, which could inflate the false positive rate for the observed association. If we applied the most stringent Bonferroni correction method to control for multiple comparisons, the critical *P* value would be 0.002 if we counted all different cancer sites (n = 21). Under such an assumption of two-sided type I error rate *P* value, the association between blood type B and risk of carcinoma reached statistical significance level. However, our hypothesis that the blood type A is associated with high risk of cancer in gastrointestinal tract is supported by potential biological mechanisms and previous studies, thus it may not require for such a stringent criterion. Another limitation is only male participants with a narrow age range, thus the findings may not be directly applicable to the general population. Studies conducted among other populations, e.g., women and/or younger age groups, are needed to replicate the study findings.

In summary, the present study demonstrates the variation in risk of carcinoma and adenocarcinoma by different ABO blood types. Compared with blood type A, Chinese men with blood type B were at statistically significantly lower risk of gastrointestinal cancers and bladder cancer while those with blood type AB were at increased risk of liver cancer.

## Supporting information

S1 Table Hazard ratios (95% confidence intervals) for cancer associated with ABO blood type, Shanghai Cohort Study 1986–2013, excluding cancer cases and person-years occurred within two years of blood draw. (DOCX)

S1 File Data. (SAS7BDAT)

## Abbreviations

CI: confidence interval
HR: hazard ratio
ICD-O: International Classification of Diseases for Oncology
