# Supplementary material for: ABO blood type and the risk of cancer – Findings from the Shanghai Cohort Study
Source: PLoS One. 2017 Sep 7;12(9):e0184295. doi: 10.1371/journal.pone.0184295 (PMC5589178; doi:10.1371/journal.pone.0184295)
Supplement: S1 Table — (DOCX) [file pone.0184295.s001.docx]

**Supplemental Table 1. Hazard ratios (95% confidence intervals) for cancer associated with ABO blood type, Shanghai Cohort Study 1986-2013, excluding cancer cases and person-years occurred within two years of blood draw ^a^**

|  | ABO blood type | | | |
| --- | --- | --- | --- | --- |
|  | A | B | AB | O |
|  |  |  |  |  |
| All cancer | 1.00 (referent) | **0.92 (0.84-1.00) ^b^** | 0.92 (0.82-1.04) | 0.95 (0.88-1.03) |
|  |  |  |  |  |
| By cancer site |  |  |  |  |
| Digestive system | 1.00 (referent) | **0.89 (0.79-1.00) ^b^** | 0.86 (0.73-1.02) | 0.93 (0.83-1.03) |
| Gastrointestinal tract | 1.00 (referent) | **0.82 (0.71-0.94) ^b^** | **0.71 (0.58-0.88) ^b^** | 0.89 (0.78-1.02) |
| Esophagus | 1.00 (referent) | 1.16 (0.75-1.78) | 0.65 (0.32-1.34) | 0.91 (0.59-1.42) |
| Stomach | 1.00 (referent) | **0.75 (0.60-0.94) ^b^** | 0.77 (0.56-1.05) | 0.86 (0.70-1.06) |
| Colorectum | 1.00 (referent) | **0.80 (0.65-0.98) ^b^** | **0.66 (0.48-0.90) ^b^** | 0.85 (0.70-1.04) |
| Liver | 1.00 (referent) | 1.04 (0.77-1.41) | **1.55 (1.09-2.21) ^b^** | 1.19 (0.90-1.57) |
| Pancreas | 1.00 (referent) | 0.97 (0.65-1.45) | 0.75 (0.41-1.38) | 0.86 (0.58-1.29) |
| Respiratory system | 1.00 (referent) | 1.00 (0.84-1.17) | 1.04 (0.83-1.29) | 0.97 (0.82-1.13) |
| Lung and trachea | 1.00 (referent) | 0.97 (0.82-1.15) | 1.01 (0.80-1.27) | 0.96 (0.82-1.13) |
| Bone/connective tissue/skin | 1.00 (referent) | 0.97 (0.53-1.77) | 0.39 (0.12-1.29) | 0.76 (0.41-1.40) |
| Genitourinary organs | 1.00 (referent) | 0.81 (0.64-1.03) | 0.90 (0.65-1.23) | 0.91 (0.73-1.13) |
| Prostate | 1.00 (referent) | 0.88 (0.64-1.21) | 0.88 (0.56-1.38) | 0.81 (0.59-1.10) |
| Urinary bladder | 1.00 (referent) | 0.66 (0.43-1.02) | 1.04 (0.61-1.75) | 1.04 (0.72-1.50) |
| Lymphoma/multiple myeloma/leukemia | 1.00 (referent) | 0.94 (0.63-1.41) | 0.87 (0.49-1.55) | 1.03 (0.70-1.51) |
| All other or unspecified sites | 1.00 (referent) | 1.21 (0.79-1.85) | 1.52 (0.90-2.58) | 1.44 (0.97-2.14) |
|  |  |  |  |  |
| By cancer histology |  |  |  |  |
| Carcinoma | 1.00 (referent) | **0.84 (0.75-0.94) ^b^** | **0.81 (0.69-0.94) ^b^** | 0.93 (0.84-1.03) |
| Epidermoid carcinoma | 1.00 (referent) | 0.90 (0.72-1.11) | 0.76 (0.55-1.04) | 0.96 (0.79-1.18) |
| Adenocarcinoma | 1.00 (referent) | **0.84 (0.74-0.96) ^b^** | **0.81 (0.67-0.98) ^b^** | 0.89 (0.78-1.01) |
| Sarcoma/lymphoma/leukemia | 1.00 (referent) | 0.96 (0.64-1.42) | 0.78 (0.52-1.16) | 0.77 (0.43-1.39) |
| All other/unspecified neoplasma | 1.00 (referent) | 1.07 (0.92-1.23) | 1.17 (0.97-1.41) | 1.02 (0.89-1.17) |

^a^ Adjusted for age at baseline, body mass index, year of interview, level of education, smoking status (never, former, current), and alcohol intake (nondrinker, <3 drinks/day, 3+ drinks/day).

^b^ Two-sided P < 0.05.
